# *Mek1*^Y130C^ mice recapitulate aspects of human cardio-facio-cutaneous syndrome

**DOI:** 10.1242/dmm.031278

**Published:** 2018-03-01

**Authors:** Rifdat Aoidi, Nicolas Houde, Kim Landry-Truchon, Michael Holter, Kevin Jacquet, Louis Charron, Suguna Rani Krishnaswami, Benjamin D. Yu, Katherine A. Rauen, Nicolas Bisson, Jason Newbern, Jean Charron

**Affiliations:** 1 Centre de recherche sur le cancer de l'Université Laval, CRCHU de Québec, L'Hôtel-Dieu de Québec, Québec G1R 3S3, Canada; 2 Department of Molecular Biology, Medical Biochemistry and Pathology, Université Laval, Québec G1V 0A6, Canada; 3 School of Life Sciences, Arizona State University, Tempe, AZ 85281, USA; 4 Institute for Genomic Medicine, Division of Dermatology, University of California San Diego, La Jolla, CA 92093-0761, USA; 5 Interpreta Inc., San Diego, CA 92121, USA; 6 Department of Pediatrics, Division of Genomic Medicine, University of California Davis, Sacramento, CA 95817, USA

**Keywords:** Cardio-facio-cutaneous syndrome, MEK1 Y130C mutation, Mouse model, Pulmonary artery stenosis, RAS/MAPK pathway, Neurological defects

## Abstract

The RAS/MAPK signaling pathway is one of the most investigated pathways, owing to its established role in numerous cellular processes and implication in cancer. Germline mutations in genes encoding members of the RAS/MAPK pathway also cause severe developmental syndromes collectively known as RASopathies. These syndromes share overlapping characteristics, including craniofacial dysmorphology, cardiac malformations, cutaneous abnormalities and developmental delay. Cardio-facio-cutaneous syndrome (CFC) is a rare RASopathy associated with mutations in *BRAF*, *KRAS*, *MEK1* (*MAP2K1*) and *MEK2* (*MAP2K2*). *MEK1* and *MEK2* mutations are found in ∼25% of the CFC patients and the *MEK1*^Y130C^ substitution is the most common one. However, little is known about the origins and mechanisms responsible for the development of CFC. To our knowledge, no mouse model carrying RASopathy-linked *Mek1* or *Mek2* gene mutations has been reported. To investigate the molecular and developmental consequences of the *Mek1*^Y130C^ mutation, we generated a mouse line carrying this mutation. Analysis of mice from a *Mek1* allelic series revealed that the *Mek1*^Y130C^ allele expresses both wild-type and Y130C mutant forms of MEK1. However, despite reduced levels of MEK1 protein and the lower abundance of MEK1 Y130C protein than wild type, *Mek1*^Y130C^ mutants showed increased ERK (MAPK) protein activation in response to growth factors, supporting a role for MEK1 Y130C in hyperactivation of the RAS/MAPK pathway, leading to CFC. *Mek1*^Y130C^ mutant mice exhibited pulmonary artery stenosis, cranial dysmorphia and neurological anomalies, including increased numbers of GFAP^+^ astrocytes and Olig2^+^ oligodendrocytes in regions of the cerebral cortex. These data indicate that the *Mek1*^Y130C^ mutation recapitulates major aspects of CFC, providing a new animal model to investigate the physiopathology of this RASopathy.

This article has an associated First Person interview with the first author of the paper.

## Abstract (precis)

**Summary:** A mouse model for cardio-facio-cutaneous syndrome caused by MEK1 Y130C mutant protein reveals the role of hyperactivation of the RAS/MAPK pathway in the development of the syndrome.

## INTRODUCTION

The RAS/MAPK signaling pathway is one of the best-characterized signaling systems, owing to its regulation of various cellular processes, including proliferation, differentiation, survival and cell death (Shaul and Seger, 2007; Sun et al., 2015). Somatic deregulation of the RAS/MAPK pathway is among the primary causes of cancer, leading this pathway to be heavily studied in the context of oncogenesis. Phases II and III studies have tested MEK (MAP2K) inhibitors for treatment of several tumor types, including breast, colon, endometrial, melanoma and non-small cell lung cancers (Catalanotti et al., 2013; Coleman et al., 2015; Dhillon, 2016; Flaherty et al., 2012; Haura et al., 2010; Lugowska et al., 2015; Rinehart et al., 2004; Signorelli and Shah Gandhi, 2016).

Germline mutations in genes encoding members of the RAS/MAPK pathway also cause developmental syndromes, such as neurofibromatosis type 1 (NF1), Noonan syndrome (NS), Costello syndrome (CS), cardio-facio-cutaneous syndrome (CFC), LEOPARD syndrome and Legius syndrome, all grouped under the appellation of RASopathies (Aoki et al., 2016; Jindal et al., 2015; Rauen, 2013; Rauen et al., 2015). These syndromes share many characteristics, such as craniofacial dysmorphology, cardiac malformations, cutaneous abnormalities and neurocognitive delay. The first RASopathy to be described was NF1, caused by mutations in the gene coding for neurofibromin1, a RAS-GTPase-activating protein (RAS-GAP). The clinical diagnosis of NF1 is based on the presence of café-au-lait maculae. NF1 patients have variable expressivity but often show anomalies of the central nervous system (Chen et al., 2015). One of the brain abnormalities associated with NF1 is astrogliosis. Astrogliosis and astrocyte activation are common responses to brain injury, and commonly marked by upregulation of proteins such as cytokines, growth factors, transcription factors and the astrocyte intermediate filament protein glial fibrillary acidic protein (GFAP) (Pekny et al., 2014; Rizvi et al., 1999; Sofroniew and Vinters, 2010). During the past decade, additional genes involved in RASopathies have been identified. For example, NS is associated with mutations in *A2ML1*, *CBL*, *KRAS*, *LZTR1*, *MAP3K8*, *MYST4*, *NRAS*, *PTPN11*, *RAF1*, *RASA2*, *RRAS*, *RIT1*, *SHOC2*, *SOS1*, *SOS2* and *SPRY1*, whereas mutations in *HRAS* and *SPRED1* have been described for CS and Legius syndrome, respectively (Jindal et al., 2015; Tidyman and Rauen, 2016a,b).

CFC is a rare syndrome with a prevalence of 1/810,000 in Japan (Orphanet; www.orpha.net/consor/cgi-bin/index.php). To date, a few hundred cases have been reported worldwide (Roberts et al., 2006; Seth et al., 2016). CFC patients have multiple congenital abnormalities that overlap with NS and CS, including craniofacial defects, hypertrophic cardiomyopathy, pulmonary artery stenosis, neurological defects and neurocognitive delay (Roberts et al., 2006). Four genes have been associated with CFC: *BRAF*, *KRAS*, *MEK1* (*MAP2K1*) and *MEK2* (*MAP2K2*). Mutations in *BRAF* correspond to ∼75% of the cases, whereas *MEK1* and *MEK2* mutations are found in ∼25% of patients (Dentici et al., 2009; Niihori et al., 2006; Rodriguez-Viciana et al., 2006). MEK1 and MEK2 are dual-specificity serine/threonine and tyrosine kinases responsible for ERK1 (MAPK3) and ERK2 (MAPK1) activation. Most MEK mutations in CFC individuals are missense, but cases carrying a *Mek2* deletion have also been reported (Dentici et al., 2009; Nowaczyk et al., 2014).

Mouse models have been generated that recapitulate genetic defects observed in CFC, but all focused on *BRAF* mutations. The first CFC mouse model expresses low levels of the oncogenic BRAF V600E protein (*B-Raf^+/^*^LSLV600E^ mutants), a constitutively BRAF active form linked to cancer, but not reported in CFC patients (Urosevic et al., 2011). It recapitulated three of the major CFC symptoms: facial dysmorphia, cardiomegaly and epileptic seizures. In parallel, mice carrying a *BRAF* L597V mutation detected in CFC patients also showed CFC characteristics: short stature, facial dysmorphia and cardiac enlargement (Andreadi et al., 2012). A third mouse model carrying the most prevalent CFC mutation, *BRAF* Q241R, showed embryonic skeletal abnormalities, lymphatic defects, cardiac defects and liver necrosis (Inoue et al., 2014). Despite these existing models, little is known about when and how CFC phenotypes develop and progress (Rodriguez-Viciana and Rauen, 2008).

No mouse model carrying CFC mutations in the *Mek1* or *Mek2* genes has been reported. Of *MEK1* and *MEK2* mutations in humans, the *MEK1*^Y130C^ mutation is the most common (Nava et al., 2007; Tidyman and Rauen, 2016a,b). To investigate the molecular and developmental effects of this mutation, we targeted the MEK1 Y130C point mutation to the third exon of the endogenous *Mek1* gene. Heterozygous *Mek1*^+/Y130C^ mice were found viable and fertile. While characterizing the *Mek1*^Y130C^ mutation, we identified an intragenic duplication produced by unequal crossing over. The *Mek1*^Y130C^ mutant allele produced both endogenous wild-type (wt) MEK1 and MEK1 Y130C proteins. The MEK1 Y130C mutant protein was more active than endogenous MEK1 and produced augmented levels of phosphorylated ERK protein in response to growth factors. Moreover, all *Mek1*^+/Y130C^, *Mek1*^Y130C/−^ and *Mek1*^Y130C/Y130C^ mice presented cranial, neurological and cardiac phenotypes similar to those observed in CFC individuals. This supports a role for deregulation of the RAS/MAPK pathway in development of CFC. Our study is the first to report a CFC mouse model carrying a *Mek1* mutation.

## RESULTS

### Viability of *Mek1*^Y130C^ mice

**Fig. 1.**
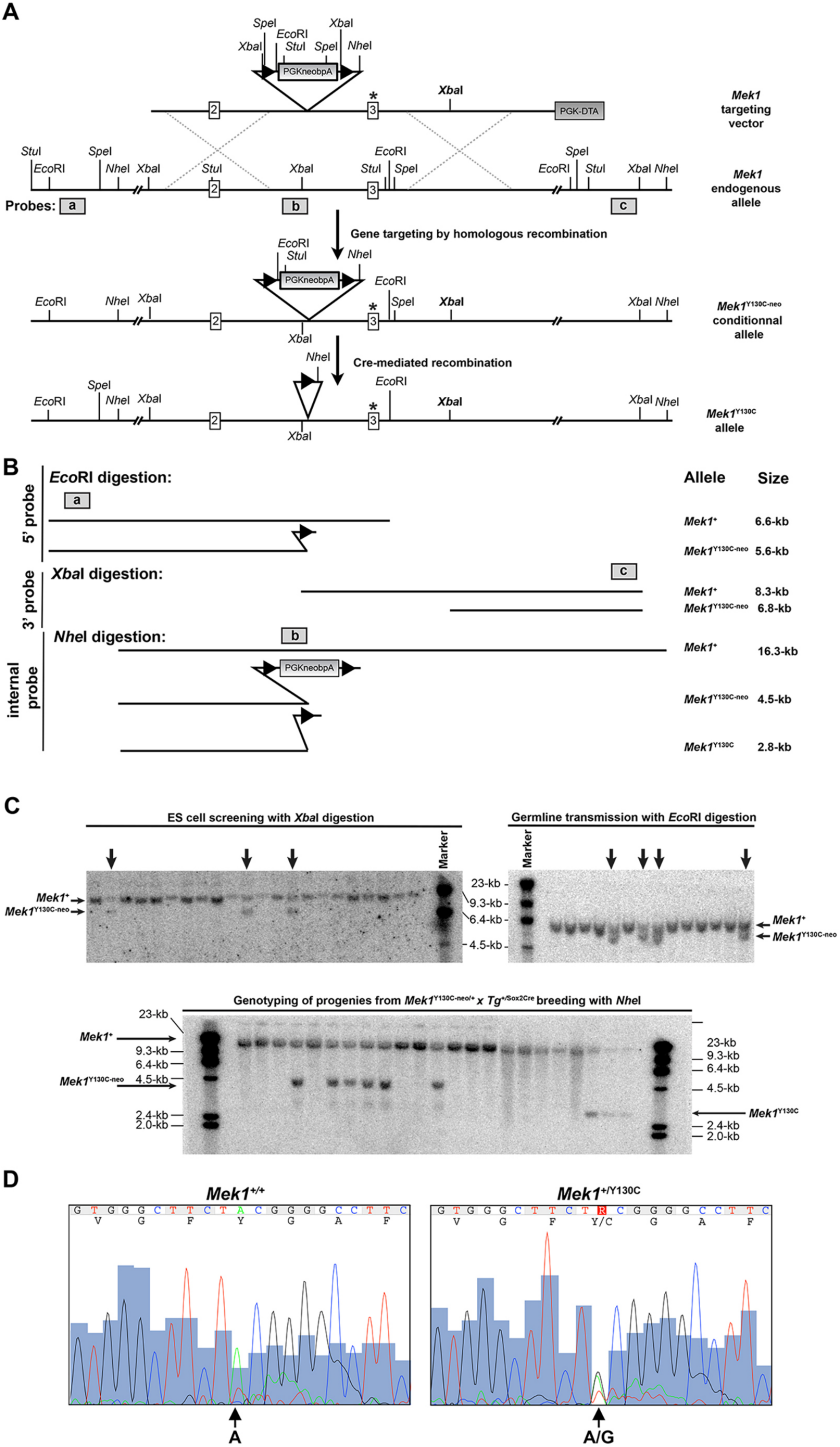
**Generation of the *Mek1*^Y130C^ allele.** (A) *Mek1* gene-targeting strategy for the generation of the Y130C point mutation. Exons are represented as white boxes. The targeting vector contains a *PGKneo* selection cassette flanked by *loxP* sites (black triangles), a PGK-DTA selection cassette (gray box), an A to G substitution in exon 3 (asterisk), and a new *Xba*I site (in bold) inserted into the third intron to facilitate the identification of the targeted allele by Southern blot analysis. Insertion of the targeting vector by homologous recombination will generate the *Mek1*^Y130C-neo^-targeted allele. Cre-mediated recombination of the *Mek1*^Y130C-neo^ allele will generate the *Mek1*^Y130C^ allele. Location of the 5′, internal and 3′ probes used for Southern analyses is indicated as gray boxes (a,b,c, respectively). (B) Schematic representation of DNA fragments obtained after *Eco*RI, *Xba*I and *Nhe*I digestions detected by Southern analyses with the 5′, 3′ and internal probes, respectively. (C) Southern blot analyses for ES cell screening (left panel; *Xba*I digestion with probe c), germline transmission (right panel; *Eco*RI digestion with probe a) and Cre-mediated deletion (lower panel; *Nhe*I digestion with probe b). The position of the different alleles is indicated on the side of the gels. (D) Sequence analysis of amplified exon 3 fragment from *Mek1^+/+^* (left panel) and *Mek1*^+/Y130C^ (right panel) specimens, confirming the presence of the A to G transition mutation, leading to the substitution of a tyrosine for a cysteine.

We designed a targeting vector carrying an A to G substitution in the *Mek1* third exon to introduce the conditional Y130C mutation into the MEK1 protein (Fig. 1A). The *neo* selection cassette flanked by *loxP* sites was inserted in the second intron of *Mek1*, generating a null allele, owing to the presence of a polyadenylation signal after the *neo* sequences that interrupts the transcription of the *Mek1* gene. Cre-mediated deletion of the *neo* sequences allowed the production of the *Mek1*^Y130C^ allele. An *Xba*I site was inserted into the third intron to facilitate the identification of the targeted allele by Southern blot analysis (Fig. 1A). The *Xba*I digestion, followed by hybridization with a 3′ probe (probe c; Fig. 1A), generated 8.3 and 6.6 kb fragments for the endogenous and targeted alleles, respectively (Fig. 1B,C). Germline transmission of the *Mek1*^Y130C-neo^ allele was verified by Southern blot analysis using an *Eco*RI digestion that distinguished the wt allele (*Mek1*^+^; 6.6 kb) and the *Mek1*^Y130C-neo^ allele (5.6 kb) with a 5′ probe (probe a; Fig. 1B,C). One germline transmitter was obtained and used to establish the *Mek1*^+/Y130C-neo^ line.

To assess the viability of *Mek1*^+/Y130C^ mutants, *Mek1*^+/Y130C-neo^ mice were bred to *Sox2Cre* mice to remove the *PGKneopA* cassette and generate the *Mek1*^Y130C^ allele. Southern blot analysis, using *Nhe*I digestion and the internal probe b, identified the *Mek1*^Y130C^ allele, which corresponded to a 2.8 kb fragment, compared to the 16.3 and 4.5 kb fragments associated with the wt and *Mek1*^Y130C-neo^ alleles, respectively (Fig. 1C). The Mendelian ratio of *Mek1*^+/Y130C^ mice was obtained at weaning (Table 1A). Moreover, normal transmission of the *Mek1*^Y130C^ allele was observed when *Mek1*^+/Y130C^ mice were crossed to *Mek1*^+/+^ animals, indicating that *Mek1*^+/Y130C^ mice were viable and fertile (Table 1B). Genomic DNA from *Mek1*^+/Y130C^ mice was used to amplify the third exon of *Mek1* for sequence analysis. This confirmed that both *Mek1*^+^ and *Mek1*^Y130C^ alleles, the latter with the A to G transition in *Mek1* third exon, were present in *Mek1*^+/Y130C^ mutants (Fig. 1D).

**Table 1.**
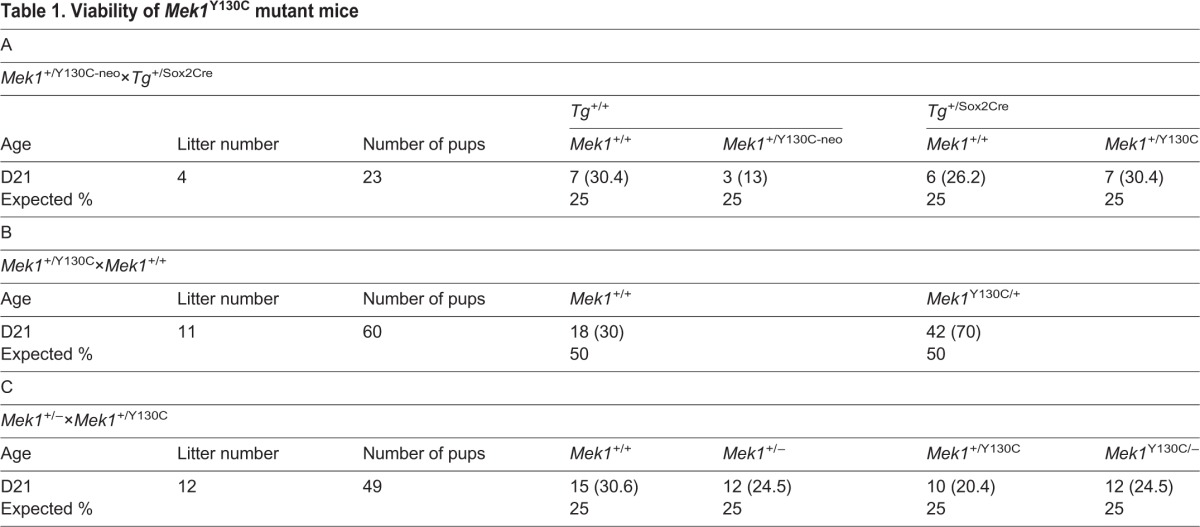
**Viability of *Mek1*^Y130C^ mutant mice**

### The *Mek1*^Y130C^ allele contains a partial duplication of the *Mek1* gene

**Fig. 2.**
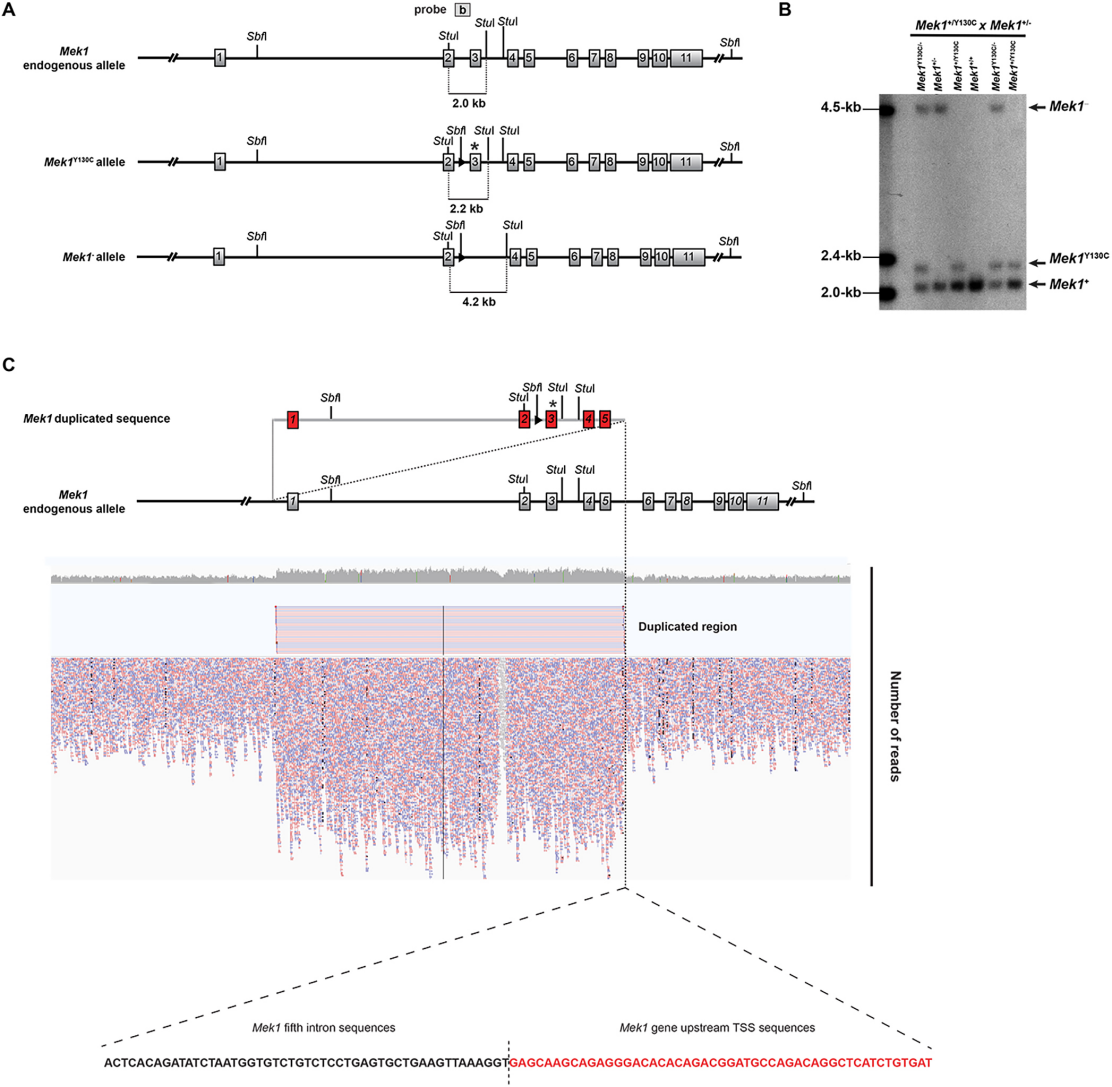
**The *Mek1*^Y130C^ allele contains a partial duplication of the *Mek1* gene.** (A) Schematic of the endogenous *Mek1* gene, the *Mek1*^Y130C^ allele and the *Mek1* null allele with the expected DNA fragments after *Stu*I digestion and detection with probe b. (B) Southern blot analysis of tail DNA from a litter obtained following *Mek1*^+/−^×*Mek1*^+/Y130C^ breeding. DNA was digested with *Stu*I, blotted and hybridized with probe b. Mice positive for the *Mek1*^Y130C^ allele always carried a wt allele even when a null allele was detected. The wt allele showed a stronger signal equivalent to the one of wt mice, suggesting *Mek1* gene duplication in the *Mek1*^Y130C^ allele. (C) Whole-genome sequencing alignment reveals a 61.5 kb *Mek1* duplication starting 6.5 kb upstream of the *Mek1* transcription start site and ending in the fifth intron. Only one break point was observed between the fifth intron in 5′ and *Mek1* promoter sequences in 3′, suggesting intragenic duplication occurring by unequal crossing over.

To investigate the biochemical properties of the *Mek1*^Y130C^ allele *in vitro*, we intercrossed *Mek1*^+/−^ mice with *Mek1*^+/Y130C^ mice to generate mouse embryonic fibroblasts (MEFs) from embryonic day (E) 13.5 *Mek1*^Y130C/−^ embryos to circumvent the absence of a MEK1 Y130C-specific antibody. *Mek1*^+^, *Mek1*^Y130C^ and *Mek1*^−^ null alleles were resolved by Southern blot analysis as *Stu*I fragments of 2.0, 2.2 and 4.2 kb, respectively (Fig. 2A). Unexpectedly, this breeding produced animals carrying the three *Mek1* alleles (Fig. 2B). Moreover, *Mek1*^+/Y130C^ specimens showed a stronger signal for the *Mek1*^+^ allele than for the *Mek1*^Y130C^ allele. In *Mek1*^+/Y130C^ mutants, the *Mek1*^+^ allele signal was similar to that observed in *Mek1*^+/+^ specimens, suggesting that duplication of the *Mek1* gene might have happened when generating the *Mek1*^Y130C^ allele. We thus examined whether duplication occurred on mouse chromosome 9, and whether it encompassed the entire *Mek1* gene. First, we performed a whole-genome sequencing analysis of DNA from *Mek1*^Y130C/Y130C^ MEFs. This revealed a 61.4 kb duplication that initiates 5.6 kb upstream of the *Mek1* transcription start site (TSS) of *Mek1* gene and finishes in the fifth intron, with equal representation of wt and Y130C sequences (Fig. 2C). A single break junction was detected, located between the fifth intron in the 5′ copy and the upstream sequence of *Mek1* locus in the 3′ copy (Fig. 2C; Table S1). These data established that an intragenic duplication occurred due to unequal crossing over.

The next generation whole-genome sequencing did not distinguish whether the Y130C mutation was in the 5′ or 3′ duplication of the *Mek1*^Y130C^ allele. To define this, we took advantage of the *Sbf*I site located in the *loxP* sequence introduced in the second intron of the Y130C copy. If the Y130C mutation is present in the 5′ duplication, the *Sbf*I digestion using a probe located in the fifth intron will generate two fragments of 70 and 26 kb in Southern blot analysis. Conversely, if the mutation is located in the 3′ duplication, two DNA bands of 61 and 35 kb will be produced. The wt allele will generate a 70 kb band (Fig. S1A). As expected, the 70 kb DNA fragment was detected in the wt specimen, whereas fragments of 70 and 26 kb were obtained in the *Mek1*^Y130C/Y130C^ sample (Fig. S1B). Thus, the *Mek1*^Y130C^ allele contained a duplication generated by unequal crossing over and the Y130C point mutation was located in the 5′ duplication.

### The *Mek1*^Y130C^ allele produces the MEK1 Y130C protein *in vivo*

**Fig. 3.**
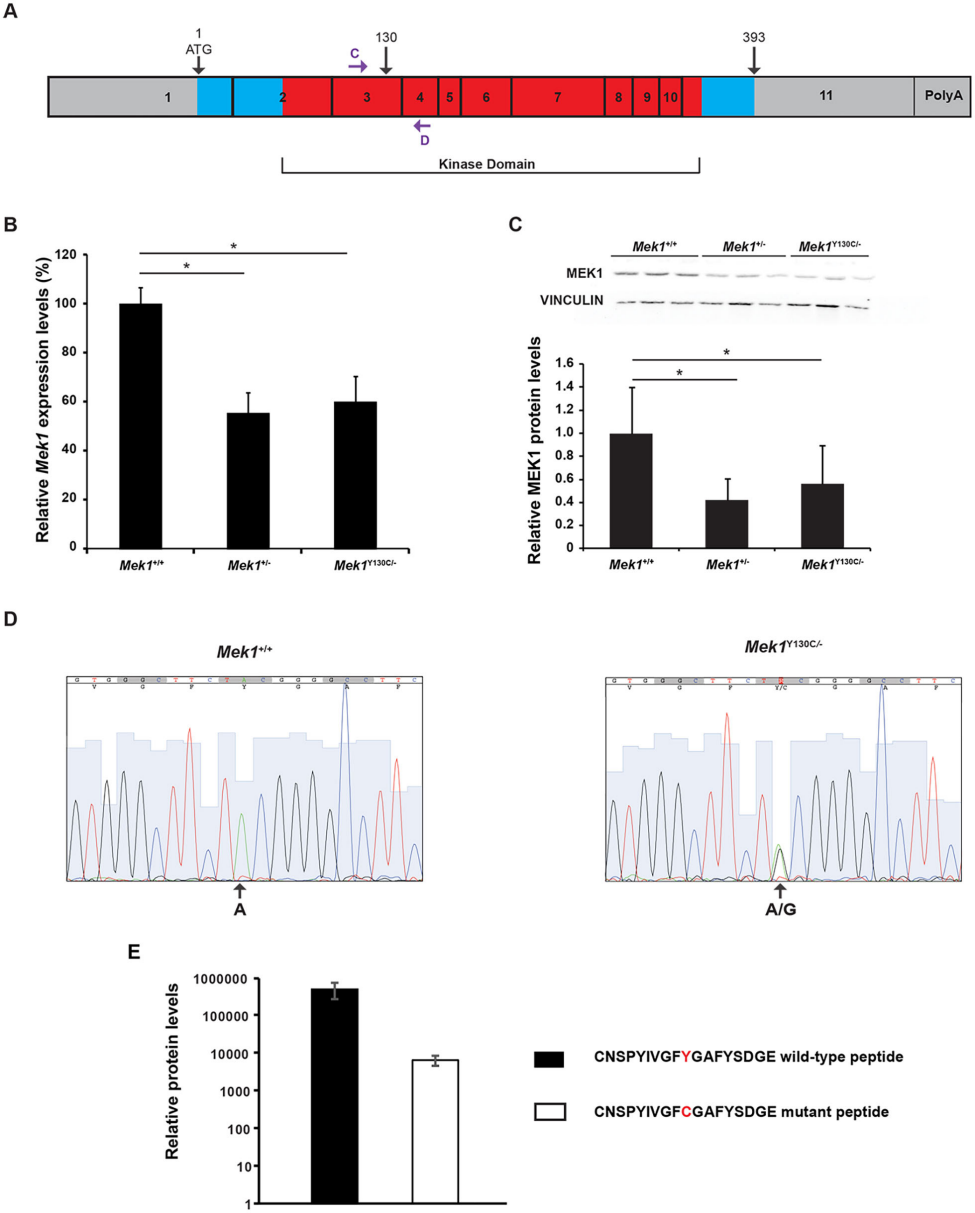
**MEK1 Y130C expression levels.** (A) Schematic representation of *Mek1* mRNA with the eleven exons. Blue and red boxes represent the translated region (amino acids 1 to 393) and the kinase domain, respectively. The position of the Y130C point mutation is indicated, as well as that of primers C and D used for qRT-PCR. (B) *Mek1* mRNA expression levels were assessed by qRT-PCR analysis on RNA isolated from *Mek1^+/+^* (*n*=4), *Mek1*^+/−^ (*n*=4) and *Mek1*^Y130C/−^ (*n*=6) kidneys. (C) MEK1 protein levels were evaluated by western blot analysis of total protein extracts from *Mek1^+/+^* (*n*=3), *Mek1*^+/−^ (*n*=3) and *Mek1*^Y130C/−^ (*n*=3) kidneys. Vinculin was used as a loading control. Quantification showed a significant diminution of *Mek1* mRNA and MEK1 protein levels in *Mek1^+/−^* and *Mek1*^Y130C/−^ mutants compared to *Mek1^+/+^* specimens. Values are reported as mean±s.e.m. (D) qRT-PCR products obtained from *Mek1*^+/+^ and *Mek1*^Y130C/−^ samples analyzed in B were sequenced. Sequence from *Mek1*^Y130C/−^ samples showed equal representation of A and G at the mutation site, indicating the presence of transcript encoding the Y130C mutation. (E) Relative wt MEK1 and MEK1 Y130C protein levels were quantified by PRM-targeted mass spectrometry using the CNSPYIVGFYGAFYSDGE wt and CNSPYIVGFCGAFYSDGE Y130C peptides (mean±s.d.; *n*=2 biological replicates).

The genomic organization of the duplication should allow the production of an unspliced heteronuclear transcript containing the duplicated exons because the sole polyadenylation signal was present after *Mek1* exon 11 (Fig. S1A). By differential splicing, both *Mek1*^+^ and *Mek1*^Y130C^ transcripts could be generated from the *Mek1*^Y130C^ allele. Alternatively, only the 3′ duplication, which encodes the consecutive 11 *Mek1* exons initiated from the interrupted *Mek1* promoter, could generate a functional transcript and the MEK1 protein. To determine whether both transcripts were produced, quantitative RT-PCR (qRT-PCR) using oligo (dT) for reverse transcription and primers located in the third and fourth exons was performed to establish *Mek1* expression levels in *Mek1*^+/+^ and *Mek1*^Y130C/−^ kidneys, lungs and thymus (Fig. 3A). Cumulative (wt and Y130C) *Mek1* transcript levels in *Mek1*^Y130C/−^ tissues were roughly half those found in *Mek1*^+/+^ specimens (Fig. 3B; data not shown). Moreover, western blot analyses showed that MEK1 protein levels in *Mek1*^Y130C/−^ and *Mek1*^+/−^ mutants were reduced by half when compared to wt specimens, indicating a correlation between transcript and protein levels (Fig. 3C). qRT-PCR products were sequenced and both wt and mutant transcripts were detected in *Mek1*^Y130C/−^ specimens (Fig. 3D; data not shown). Overall, wt levels of RNA and protein were produced from the Y130C allele. This suggested that the duplication did not lead to a RNA product that is degraded.

To quantify the relative abundance of wt and MEK1 Y130C proteins, MEK1 proteins were immunoprecipitated from *Mek1*^Y130C/−^ MEF protein extracts and analyzed by parallel reaction monitoring (PRM)-targeted proteomics (Gallien et al., 2012; Peterson et al., 2012). Using isotope-labeled synthetic peptides to discriminate between MEK1 and MEK1 Y130C proteins, the analysis confirmed that both proteins were indeed present in *Mek1*^Y130C/−^ MEFs (Fig. S2). In addition, the PRM analysis clearly showed that MEK1 Y130C protein was present at lower levels when compared to wt MEK1 protein (Fig. 3E; Table S2). Altogether, these data revealed that transcription of the duplicated *Mek1*^Y130C^ allele results in transcripts that can generate both MEK1 and MEK1 Y130C proteins.

### Increased activation of the RAS/MAPK pathway in MEFs carrying the *Mek1*^Y130C^ allele

**Fig. 4.**
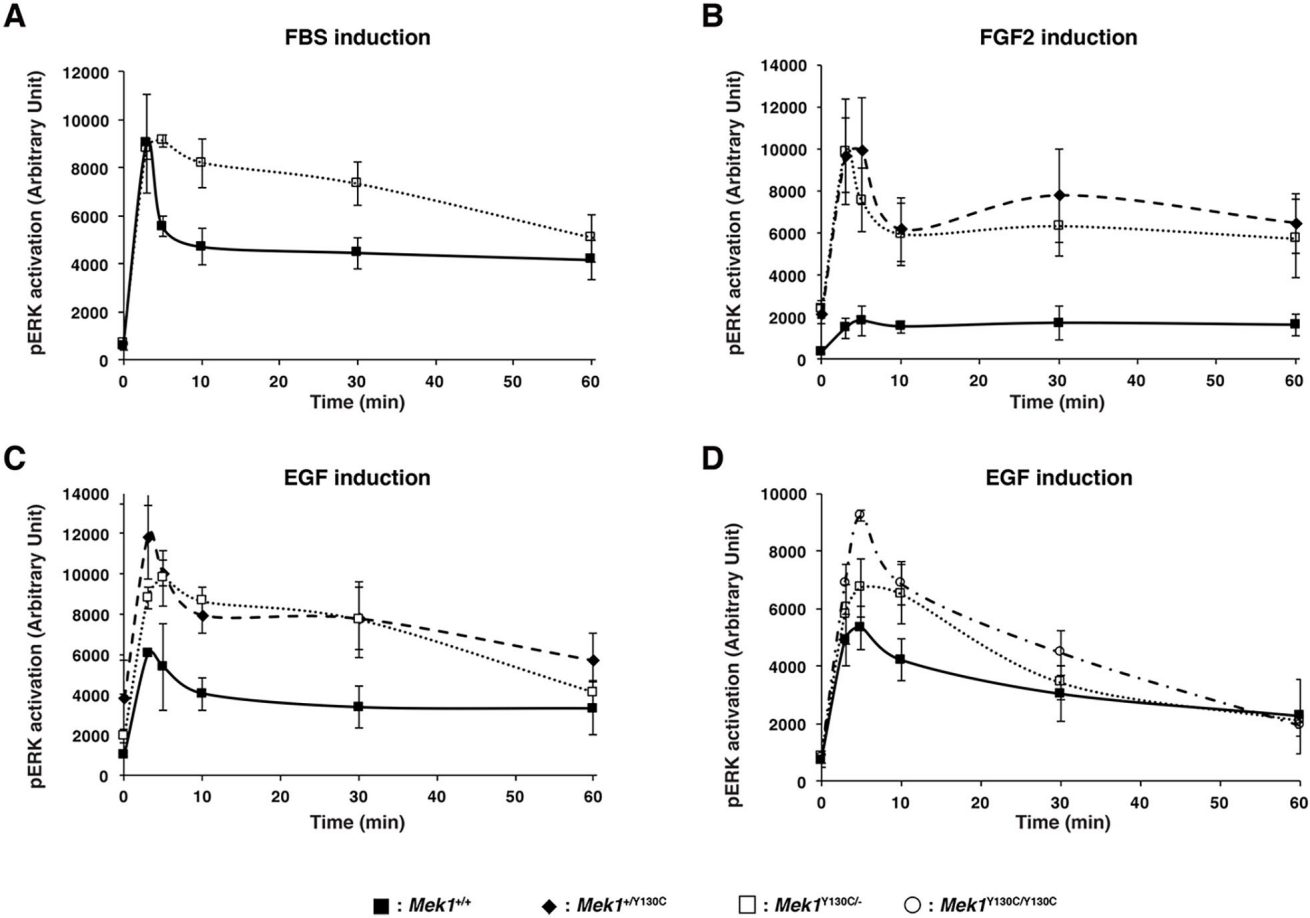
**RAS/MAPK pathway hyperactivation in MEFs carrying *Mek1*^Y130C^ allele.** (A-C) *Mek1^+/+^*-, *Mek1*^+/Y130C^- and *Mek1*^Y130C/−^-established MEF lines were stimulated with 20% FBS (in A, *Mek1*^Y130C/−^ MEFs were not tested), 2 ng/ml of FGF2 (B) or 2 ng/ml of EGF (C), and phosphorylation of ERK1/2 was assessed by quantitative immunoblotting along with total vinculin as a loading control. (D) Three *Mek1^+/+^*, *Mek1*^Y130C/−^ and *Mek1*^Y130C/Y130C^ primary MEF cultures were treated with 2 ng/ml EGF and phosphorylation of ERK1/2 was assessed. Values are reported as mean±s.e.m. in arbitrary units (*n*=3).

*In vitro* studies suggested that CFC mutations are gain-of-function mutations that hyperactivate the RAS/MAPK pathway (Rodriguez-Viciana and Rauen, 2008; Rodriguez-Viciana et al., 2006). Conversely, the CFC mouse model with a mutation in *Braf* (BRAF Q241R) developed severe phenotypes without any major impact on the activation of the RAS/MAPK pathway in whole embryo (Inoue et al., 2014). However, a slight increase in ERK phosphorylation was observed in brain samples, and treatment of these mice with MEK inhibitors partially rescued the phenotype, suggesting that the brain defects were due to hyperactivated RAS/MAPK pathway. Moreover, overexpression of a *Mek1*^Y130C^ transgene in HEK293T cells indicated that the MEK1 Y130C protein is more active than the wt form (Rodriguez-Viciana et al., 2006). To investigate the biochemical properties of the MEK1 Y130C protein, we looked at its ability to phosphorylate ERK in quiescent cells and in response to stimulation by growth factors and serum. ERK phosphorylation was quantified in wt-, *Mek1*^+/Y130C^- and *Mek1*^Y130C/−^-established MEFs stimulated with fetal bovine serum (FBS), FGF2 or EGF (Fig. 4A-C). Following serum deprivation, ERK activation in *Mek1*^+/Y130C^ and *Mek1*^Y130C/−^ MEFs showed a more elevated trend than in *Mek1^+/+^* cells. In response to FGF2 or EGF, both *Mek1*^+/Y130C^ and *Mek1*^Y130C/−^ MEFs presented similar kinetics of ERK activation, with higher levels of ERK phosphorylation than *Mek1^+/+^* cells (*P*<0.001 and *P*<0.00001 by ANOVA, respectively). To confirm that the increased phosphorylation activity was due to the MEK1 Y130C protein, we compared ERK phosphorylation in primary MEFs derived from wt, *Mek1*^Y130C/−^ and *Mek1*^Y130C/Y130C^ embryos (Fig. 4D). *Mek1*^Y130C/−^ and *Mek1*^Y130C/Y130C^ MEFs produced lower levels of MEK1 protein (wt and Y130C) when compared to wt MEFs (Fig. S3). Despite that, in response to EGF, both *Mek1*^Y130C/−^ and *Mek1*^Y130C/Y130C^ MEFs showed similar kinetics of ERK activation with higher levels of ERK phosphorylation than *Mek1^+/+^* MEFs (Fig. 4D; *P*<0.0001 by ANOVA). Thus, these results are in agreement with the notion that the MEK1 Y130C mutation generated a more active form of MEK1.

### Heart and cranial defects in *Mek1*^Y130C^ mutant mice

**Fig. 5.**
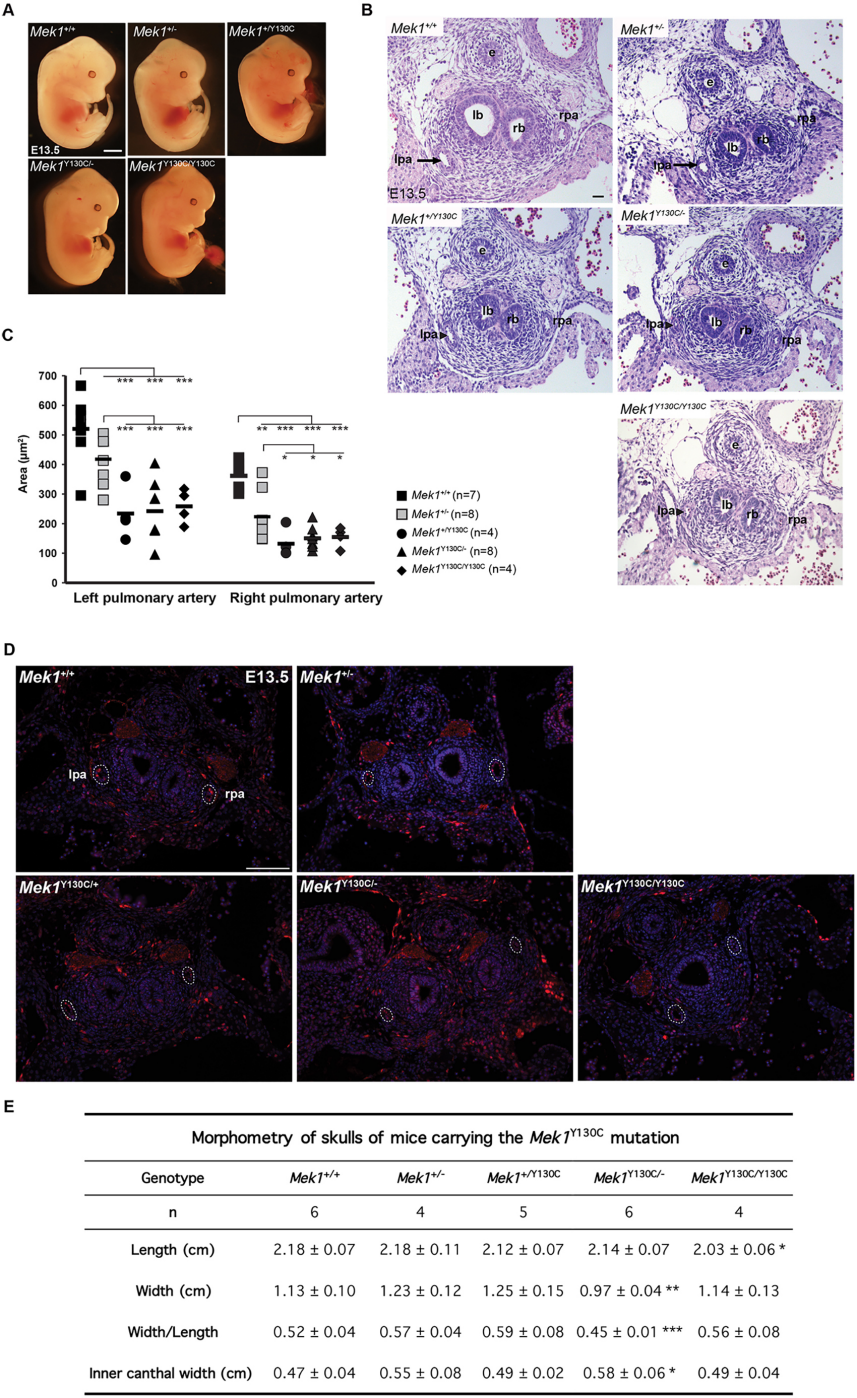
***Mek1*^Y130C^ mutant mice present cardiac and cranial anomalies.** (A) E13.5 control (*Mek1^+/+^* and *Mek^+/−^*) and *Mek1*^+/Y130C^, *Mek1*^Y130C/−^ and *Mek1*^Y130C/Y130C^ mutant embryos did not present overt morphological anomalies. (B) H&E staining of transverse sections from pulmonary arteries of E13.5 control (*Mek1^+/+^* and *Mek1*^+/−^) and *Mek1*^+/Y130C^, *Mek1*^Y130C/−^ and *Mek1*^Y130C/Y130C^ mutant embryos. Pulmonary stenosis was observed in all specimens carrying a *Mek1*^Y130C^ allele (arrowhead). e, esophagus; lb, left bronchia; lpa, left pulmonary artery (arrow); rb, right bronchia; rpa, right pulmonary artery. (C) Measurement of arterial lumen area confirmed the pulmonary stenosis for the left and right arteries in *Mek1*^+/Y130C^, *Mek1*^Y130C/−^ and *Mek1*^Y130C/Y130C^ mutants. (D) Immunostaining for phospho-ERK (pERK) was performed on sections of pulmonary arteries from E13.5 *Mek1^+/+^*, *Mek1*^+/−^ and *Mek1*^Y130C/−^ embryos. No difference was observed. (E) Morphometric characteristics of mice skulls. Length and width of the skull as well as inner canthal width were measured in cm on Alcian Blue/Alizarin Red-stained skulls. *Mek1*^Y130C/−^ mice presented facial dysmorphia with reduced skull width and increased inner canthal width. *Mek1*^Y130C/Y130C^ mutants presented reduced skull length. *P<0.05; **P<0.01; ***P<0.005. Scale bars: 25 µm (A); 100 µm (D); 200 µm (B).

All *Mek1*^+/Y130C^, *Mek1*^Y130C/−^ and *Mek1*^Y130C/Y130C^ mice were viable and fertile. To assess whether the MEK1 Y130C mutation reproduced CFC phenotypes, these three lines were compared with *Mek1^+/+^* and *Mek1^+/−^* animals. All mouse lines presented comparable length and weight at adult ages. The heart weight/body weight ratio in adult mutants was examined to determine whether they developed hypertrophic cardiomyopathy. No differences were detected. Similarly, no variation in the weight ratio was observed for the liver, kidney and spleen (data not shown). No major anomalies were observed in the gross morphology of embryos at E13.5 for the different *Mek1*^Y130C^ genotypes (Fig. 5A). Another heart defect frequently found in CFC individuals is pulmonary artery stenosis (Araki et al., 2004; Chen et al., 2010; Moriya et al., 2015). At E13.5, pulmonary artery stenosis was seen in all *Mek1*^Y130C^ mutants (Fig. 5B). The measurement of the lumen area of the pulmonary artery showed no statistical difference between *Mek1^+/+^* and *Mek1^+/−^* specimens (Fig. 5C). However, the left and right pulmonary artery lumen areas were significantly reduced in all genotypic combinations carrying a *Mek1*^Y130C^ allele when compared to controls, indicating that one *Mek1*^Y130C^ mutant allele was sufficient to cause pulmonary stenosis. To determine whether this phenotype was associated with the hyperactivation of the RAS/MAPK pathway, ERK phosphorylation was monitored by immunofluorescence staining in pulmonary arteries from E13.5 *Mek1*^+/+^, *Mek1*^+/−^, *Mek1*^Y130C/−^ and *Mek1*^Y130C/Y130C^ embryos. No variation in immunostaining was detected between the different genotypes (Fig. 5D).

CFC patients present craniofacial dysmorphia. Observation of mutant mice did not reveal obvious phenotypes. Therefore, we examined the skulls of *Mek1*^+/Y130C^, *Mek1*^Y130C/−^ and *Mek1*^Y130C/Y130C^ mutants at 6 weeks of age and compared them to *Mek1^+/+^* and *Mek1*^+/−^ specimens. Three parameters were analyzed: the length and width of the skull, and the width of the inner canthal distance (Fig. 5E). *Mek1*^Y130C/Y130C^ and *Mek1*^Y130C/−^ mutants showed significant changes in one or several of these parameters. Thus, the *Mek1*^Y130C^ allele can instigate cranial dysmorphia in mice, suggesting that it is also the cause of this phenotype in CFC patients. However, in contrast to the pulmonary artery phenotypes, which are dominantly inherited with the Y130C allele, craniofacial defects showed a lower expressivity.

### The *Mek1*^Y130C/Y130C^ mutation causes brain defects

**Fig. 6.**
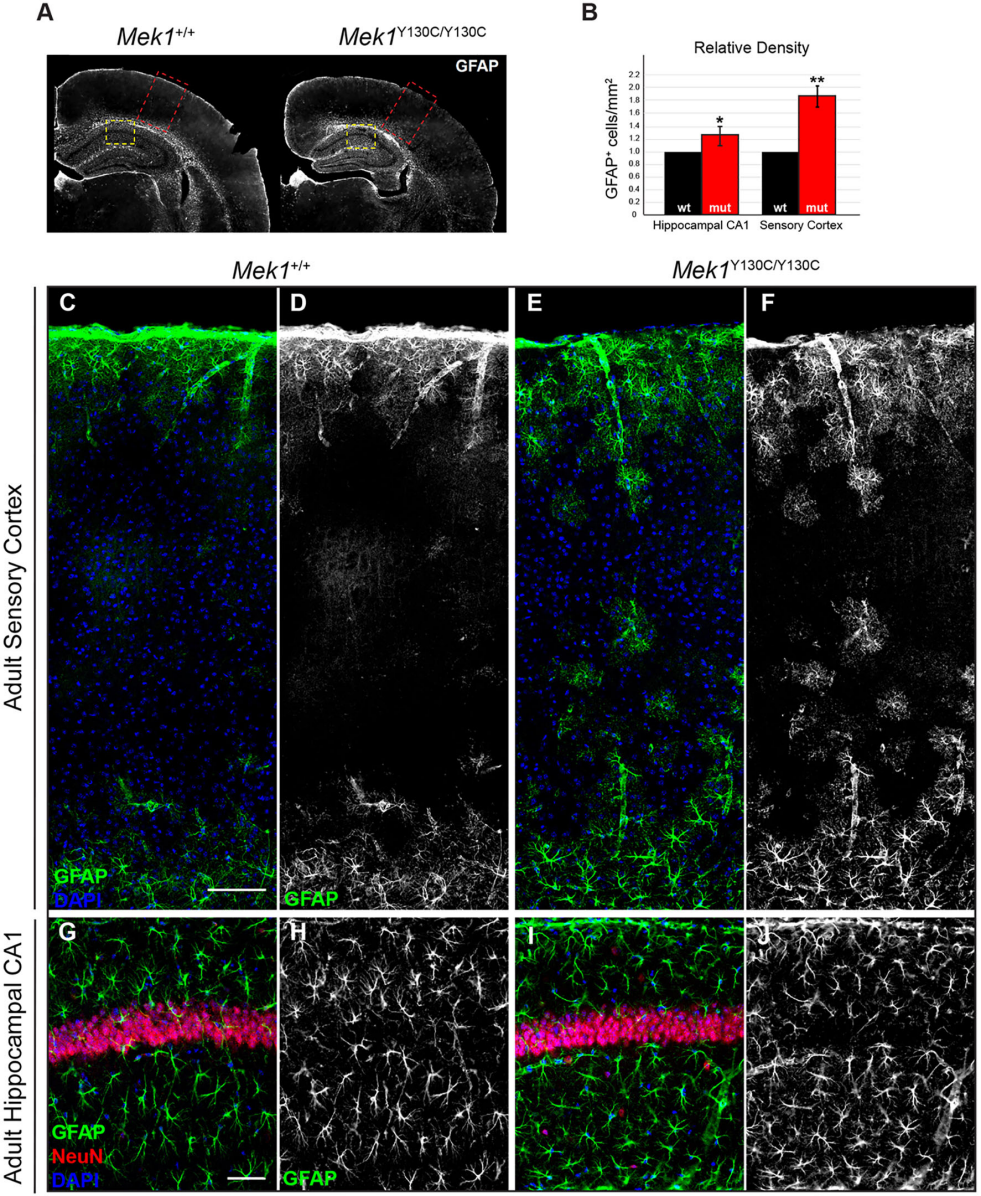
**Increased number of GFAP^+^ cells in cortical and hippocampal sections of *Mek1*^Y130C/Y130C^ mutants.** (A) Representative coronal brain sections stained for GFAP. Red dashed line boxes denote an area of sensory cortex shown in C-F; yellow dashed line boxes denote an area of hippocampal CA1 presented in G-J. (B) Quantification of relative density of GFAP^+^ cell counts is shown as a relative number of positive cells/mm^2^ (*n*=3). (C-F) *Mek1*^Y130C/Y130C^ animals exhibited an increased number of GFAP^+^ astrocytes in the sensory cortex in comparison to wt animals. (G-J) Analysis of GFAP-labeled astrocytes in hippocampal CA1 revealed a modest but significant increase in the relative density of cells/mm^2^. *P<0.05; **P<0.01. Scale bars: 50 µm (G); 100 µm (C).

**Fig. 7.**
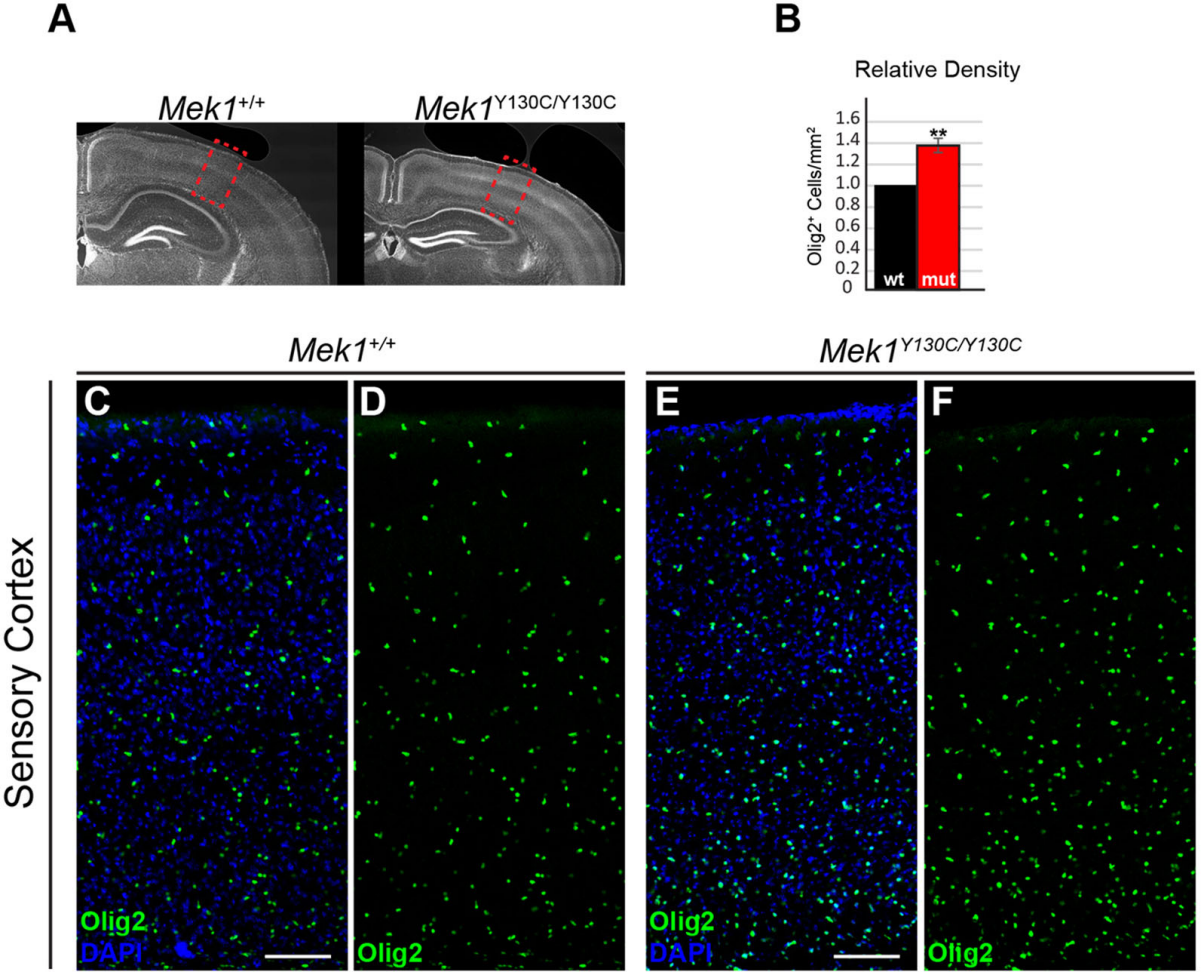
***Mek1*^Y130C/Y130C^ cortices exhibit increased density of Olig2^+^ cells.** (A) Representative coronal brain sections stained for the oligodendrocyte transcription factor (Olig2). Red boxes denote an area of sensory cortex presented in C-F. (B) Quantification of relative densities of Olig2^+^ cells in adult sensory cortex (*n*=3). (C-F) The relative density of Olig2^+^ cells was assessed in radial columns of sensory cortex. Note the increased density of Olig2^+^ cells in the mutant sensory cortex (E,F) relative to controls (C,D). **P<0.01. Scale bars: 100 µm.

Individuals with CFC exhibit a broad range of neurological abnormalities, including variable penetrance of developmental and neurocognitive delays, with ∼40% of the patients suffering from seizure disorder (Rauen, 2013; Yoon et al., 2007). Astrocytes are involved in several pathologies of the central nervous system. In general, astrogliosis, characterized by an increased GFAP expression, is known to occur in a range of neuropathological states (Middeldorp and Hol, 2011). Extensive work modeling RASopathy-associated mutations in mice has revealed numerous cellular defects in distinct neuronal and glial subtypes that might contribute to these neurological phenotypes (Anastasaki and Gutmann, 2014; Brown et al., 2012; Gauthier et al., 2007; Lee et al., 2014; Li et al., 2012; Lush et al., 2008; Paquin et al., 2009; Pucilowska et al., 2012; Xing et al., 2016). A cellular abnormality frequently observed in postmortem tissues from NF1 patients and in NF1 mouse models is an increased number of GFAP^+^ astrocytes in cortical and hippocampal gray matter (Gutmann et al., 1999; Hegedus et al., 2007; Nordlund et al., 1995; Rizvi et al., 1999; Zhu et al., 2005). The relative number of GFAP^+^ astrocytes in the sensory cortex and hippocampal CA1 regions was assessed in *Mek1*^Y130C/Y130C^ adults (Fig. 6A). Consistent with other RASopathy-linked mutations, an increased density of GFAP^+^ astrocytes was observed in *Mek1*^Y130C/Y130C^ mutant sensory cortices (Fig. 6B,C-F). A similar increase in GFAP^+^ astrocyte numbers in hippocampal CA1 was detected (Fig. 6B,G-J). Enhanced myelination and oligodendrocyte progenitor numbers were also reported in RASopathy models (Bennett et al., 2003; Ehrman et al., 2014; Ishii et al., 2013). The total cortical oligodendrocyte population was therefore analyzed in *Mek1*^Y130C/Y130C^ mutants by immunolabeling for Olig2, a master transcription factor essential in oligodendrocyte fate decisions (Mizuguchi et al., 2001; Novitch et al., 2001; Zhou et al., 2001). *Mek1*^Y130C/Y130C^ mice exhibited increased density of Olig2^+^ nuclei in the sensory cortex relative to littermate controls (Fig. 7). ERK activation was also assayed by phospho-ERK (pERK) immunostaining on brain sections from *Mek1*^+/+^ and *Mek1*^Y130C/Y130C^ adults. Signal was detected in the pyramidal neuron layer of the hippocampal CA1 region in both *Mek1*^+/+^ and *Mek1*^Y130C/Y130C^ specimens, and *Mek1*^Y130C/Y130C^ mutants showed an increased density of pERK-positive cells (Fig. S4). Taken together, our results demonstrated that regulation of astrocyte oligodendrocyte and pyramidal neuron populations could be an important feature in RASopathy neuropathogenesis, and further supported the validity of the *Mek1*^Y130C^ mutation as a model for CFC.

## DISCUSSION

In this study, we described the generation of a hypermorphic *Mek1* allele carrying the Y130C mutation found in a significant subset of CFC patients. The *Mek1*^Y130C^ allele contained a partial duplication of the *Mek1* gene, leading to the production from the same allele of both an endogenous MEK1 protein and the MEK1 Y130C protein at lower levels. Despite this, mice heterozygous and homozygous for the *Mek1*^Y130C^ allele present several characteristics of CFC, including one of the most frequent cardiac defects in CFC; namely, pulmonary stenosis, cranial dysmorphogenesis and neurological abnormalities (Table S3) (Roberts et al., 2006). Characterization of MEFs carrying the *Mek1*^Y130C^ allele also revealed higher ERK phosphorylation levels in response to growth factors. This increased activation of the RAS/MAPK pathway is similar to that observed in the *Raf1*^+/L613V^ and *Sos1*^+/E846K^ mouse models of NS. It supports the notion that CFC, like other RASopathies, results from abnormal hyperactivation of the RAS/MAPK pathway (Chen et al., 2010; Wu et al., 2011).

In contrast to other mouse models of RASopathies, neither heterozygous nor homozygous mice carrying the *Mek1*^Y130C^ mutation exhibit embryonic or premature lethality. Indeed, it was shown that *Braf^+/^*^Q241R^ and *B-Raf^+/^*^LSLV600E^ mutant embryos die during gestation or early after birth and present several phenotypes reminiscent of CFC (Inoue et al., 2014; Urosevic et al., 2011). In the case of *B-Raf^+/^*^LSLV600E^ mutants, perinatal survival varies depending on the genetic background. Similarly, *Raf1*^+/L613V^ and *Ptpn11*^+/D61G^ mice, as well as *K-Ras*^V14I/V14I^ homozygous mutants, which all model Noonan syndrome, display reduced (or no) survival in the C57BL/6J genetic background, whereas they are viable in a mixed background (Araki et al., 2004; Hernández-Porras et al., 2014, 2015; Wu et al., 2011). These data suggested that modifier loci might contribute to the phenotypic variation observed between RASopathy patients carrying the same allele. At least two factors might explain the milder phenotype observed in *Mek1*^Y130C^ mutant mice. First, the low levels of MEK1 Y130C protein produced by the *Mek1*^Y130C^ allele could minimize the phenotypic manifestation of CFC. Second, the 129S6 genetic background, in which the mutation was maintained, might contain modifier loci that could mask manifestations of the CFC phenotype.

Hyperactivation of the RAS/MAPK pathway in response to growth factors in cells carrying the *Mek1*^Y130C^ allele supports a role for this signaling pathway in the development of CFC. MEK1 Y130C expression was evaluated in *Mek1*^Y130C/−^ MEFs by comparing the levels of wt MEK1 and MEK1 Y130C proteins produced by the *Mek1*^Y130C^ allele using PRM. An endogenous peptide corresponding to MEK1 Y130C co-eluted with its synthetic counterpart and was clearly detected in MEK1 immuno-precipitates (Fig. S2D). The precise quantification of endogenous MEK1 Y130C levels was complicated by the interference in the quantification of the MEK1 Y130C synthetic peptide when added to total MEK1 immuno-precipitates. Tandem mass spectrometry (MS/MS) analyses confirmed the identification of the MEK1 Y130C peptide in *Mek1*^Y130C^ mutant, and evidence indicated that MEK1 Y130C protein levels are lower than those of the wt MEK1 protein. Nonetheless, in all genotypes tested, ERK phosphorylation was increased in the presence of low levels of MEK1 Y130C protein, strongly suggesting that the MEK1 Y130C is several times more active than MEK1. This is consistent with previous reported overexpression experiments (Rodriguez-Viciana and Rauen, 2008).

The genomic organization of the *Mek1*^Y130C^ allele could account for the differences in MEK1 and MEK1 Y130C protein levels. The Y130C point mutation is inserted in the 5′ duplication, implying that production of MEK1 Y130C protein requires transcription of the entire *Mek1*^Y130C^ locus to produce by alternative splicing the *Mek1*^Y130C^ full-length transcript (Fig. S1A). The production of the endogenous *Mek1* full-length transcript could proceed via the transcription from the truncated *Mek1* promoter in the 3′ duplication. qRT-PCR experiments showed that the *Mek1*^Y130C^ allele was transcribed as efficiently as the *Mek1*^+^ allele. Moreover, sequence analysis of the qRT-PCR products revealed an equivalent representation of the wt transcript and the one carrying the Y130C mutation in *Mek1*^Y130C/−^ specimens. As the sequence encompassing the third and fourth exons was the only one to be analyzed in depth, we cannot rule out the possibility that incorrect alternative splicing of the *Mek1* Y130C full-length transcript can contribute to reduced MEK1 Y130C protein levels.

The sequence duplicated in the *Mek1*^Y130C^ allele includes the *Mek1* first intron of 39 kb in which the *Uchl4* gene is located (Osawa et al., 2001). *Uchl4* is part of a gene family that encodes ubiquitin carboxyl-terminal hydrolases implicated in proteolytic processing of polymeric ubiquitin. This activity is important for cytoplasmic protein degradation and for recycling free ubiquitin via cleavage of ubiquitylated peptides produced by the proteasomal degradation of polyubiquitylated proteins (Larsen et al., 1998). Perturbation of this process may have a major impact on cytoplasmic protein degradation pathway in neurodegenerative disorders, such as Parkinson's disease. For instance, a dominant mutation in *Uchl1*, which decreases the *in vitro* hydrolytic activity of UCHL1, has been linked to higher risk of developing Parkinson's disease (Liu et al., 2002). Moreover, inactivation of *Uchl3*, the closest member of *Uchl4*, causes learning deficits in mice owing to significantly increased working memory errors (Wood et al., 2005). In both cases, the phenotype is due to a loss of function. The duplication of the *Uchl4* gene in the *Mek1*^Y130C^ allele generates a gain of function (Fig. S5). However, gain in ubiquitin carboxyl-terminal hydrolase activity might also be detrimental for protein homeostasis. More experiments are required to determine whether *Uchl4* gene duplication can contribute to the phenotypes observed in *Mek1*^Y130C^ mice.

Hyperactivation of the RAS/MAPK pathway by the MEK1 Y130C protein also supports the notion that the mutation contributes to the neurological anomalies observed in *Mek1*^Y130C/Y130C^ mutants. We have previously shown that the RAS/MAPK pathway plays a key role in gliogenesis (Li et al., 2012). Deletion of *Mek1* and *Mek2* genes in radial progenitors prevents gliogenesis, affecting astrocyte and oligodendrocyte differentiation, whereas the expression of a dominant active form of MEK1 in the same lineage leads to increased numbers of astrocytes with coincident reduction in neuron numbers. Reduced neurogenesis is consistent with the idea that hyperactive MEK accelerates radial progenitor progression into a gliogenic mode and prematurely terminates neurogenesis. Data from the *Mek1*^Y130C/Y130C^ mutants might reflect developmental defects, but they could also indicate astrogliosis in response to neurological lesion, as revealed by the increase in GFAP-positive cells in the cortex and hippocampus. Astrogliosis with accumulation of GFAP-positive cells is a defensive reaction aimed at limiting tissue damage (Stringer, 1996). Patients with CFC present with mild to severe cognitive delay, developmental delay and seizure disorder (Yoon et al., 2007). It will be interesting to test whether *Mek1*^Y130C^ mutants exhibit defects in learning ability and memory.

The *Mek1*^Y130C^ mutant mouse reported here is the first mouse model of a CFC-linked *Mek1* mutation. It provides a powerful tool to gain further understanding of the physiopathology of at least some CFC clinical features, such as pulmonary stenosis, facial defects and astrogliosis, and to investigate the associated brain defects that are more difficult to address in other CFC mouse models due to the embryonic or neonatal lethality observed.

## MATERIALS AND METHODS

### Generation of the *Mek1*^Y130C^ allele

The targeting vector was made using a 5.5 kb genomic fragment that encompasses exons 2 and 3 of the *Mek1* gene, containing 2.2 kb of 5′- and 3.3 kb of 3′-sequence homology for homologous recombination to occur. The PGK-neo selection cassette flanked by *loxP* sites was inserted in the *Bam*HI site located in *Mek1* intron 2, while the PGK-DTA negative selection cassette was located in intron 3 at the 3′-end of *Mek1* homology. An A to G transition was inserted in exon 3 to generate the MEK1 Y130C mutation (Fig. 1A). Correctly targeted ES clones were injected into MF1 blastocysts to generate chimeras as described (Bélanger et al., 2003). Chimeras were bred with 129S6 mice for transmission of the targeted *Mek1*^Y130C-neo^ allele and all subsequent breedings were done in this genetic background. *Mek1*^+/Y130C-neo^ heterozygous mice were bred with *Sox2Cre* deleter mice to generate the *Mek1*^Y130C^ allele (Hayashi et al., 2002). The *Mek1*^+/−^ mouse line was described previously (Bissonauth et al., 2006).

### Mice, genotype and tissue collection

The age of the embryos was estimated by considering the morning of the day of the vaginal plug as E0.5. Control and mutant embryos were collected at E13.5. Adult skeletons and organs were collected at 6 weeks of age. For RNA and protein extraction, organs were snap frozen in liquid N_2_. Genomic DNA from embryonic stem (ES) cells and mouse tail biopsies was extracted, purified and genotyped by Southern analysis using restriction digestions described in the text and *Mek1* genomic probes (Fig. 1B). All experiments were performed according to the guidelines of the Canadian Council on Animal Care and approved by the institutional animal care committee.

### Pulse field gel electrophoresis

MEF DNA was prepared as described (Aoidi et al., 2016). Purified DNA was digested with the *Nhe*I and *Sbf*I restriction endonucleases, fractionated by pulse field gel electrophoresis through 1% agarose gel, which was conducted in 0.5× TBE buffer (45 mM Tris-base, 1 mM EDTA, 45 mM boric acid) with a CHEF DR-II apparatus (Bio-Rad, Hercules, CA), with an initial switch time at 1 s and final switch time at 10 s for 20 h at 14°C and a voltage of 10 V/cm (Herschleb et al., 2007; Jo et al., 2013). The gel was blotted onto N-Hybond membrane (GE Healthcare Life Sciences, Mississauga, ON, Canada), and hybridized to probe d described in Fig. S1A by following procedures recommended by the supplier.

### RNA isolation and qRT-PCR

Organs were collected from wt, *Mek1*^Y130C/−^, *Mek1*^Y130C/Y130C^ and *Mek1*^−/−^ mice as described (Nadeau and Charron, 2014). Total RNA was isolated using TRIzol reagent according to the manufacturer's procedure (Life Technologies Inc., Burlington, ON). cDNA was synthesized with the Superscript II Reverse Transcriptase (Life Technologies) with 1 µg total RNA and oligo (dT). qRT-PCR experiments were performed as described using *Rpl19* gene as a control (Boucherat et al., 2012). *Mek1* primer sequences were: Forward 5′-CTGATCCACCTGGAGATCAAACC-3′ and Reverse 5′-CTCCCGAAGATAGGTCAGGC-3′.

### Western blot analysis

Protein extracts were prepared as described (Bélanger et al., 2003). Total protein lysates (20 µg) were resolved on a denaturing 10% sodium dodecyl sulfate-polyacrylamide gel electrophoresis (SDS-PAGE) and probed with rabbit monoclonal antibody against MEK1 (Clone E3442; Epitomics) and mouse monoclonal antibody against vinculin (clone hVIN-1; Sigma-Aldrich) used at 1/2000 and 1/5000, respectively (Nadeau et al., 2009). The most representative western blots are presented. The relative amount of MEK1 was obtained by densitometry analyses with the Fluor-S MAX MultiImager-captured images using ImageJ.

### Mass spectrometry

For affinity-purification of MEK1 proteins, whole cell extracts were obtained by collecting cells from 7×150 mm Petri dishes having reached 90% confluence. Cells were scraped in ice-cold lysis buffer containing freshly added protease inhibitors (20 mM Tris pH 7.4, 150 mM NaCl, 1 mM EDTA pH 8, 1% NP-40, 0.5% sodium deoxycholate, 10 mM ß-glycerophosphate, 10 mM sodium pyrophosphate, 50 mM NaF, 1 mM PMSF, 10 μg/ml leupeptin, 10 μg/ml aprotinin, 10 μg/ml pepstatin) (1 ml per 150 mm Petri) and incubated for 30 min on ice (Beigbeder et al., 2016). Samples were centrifuged for 20 min at 20,000 ***g*** and supernatants (∼20 mg of protein) were pre-cleared by incubation with 75 µl protein G sepharose beads for 90 min at 4°C. After centrifugation, supernatants were incubated with 2 µg rabbit monoclonal MEK1 antibody with rotation for 1 h at 4°C followed by another incubation for 1 h at 4°C with 75 µl protein G sepharose beads. Affinity-purified MEK1 protein was washed three times in lysis buffer and twice in ‘light’ buffer **(**20 mM Tris pH 7.4, 1 mM PMSF, 10 μg/ml leupeptin, 10 μg/ml aprotinin, 10 μg/ml pepstatin) and processed for digestion as described (Beigbeder et al., 2016), except for the digestion that was performed with 5 µg endopeptidase GluC for 2 h at 37°C, followed by a 2-h incubation at room temperature with 5 μg trypsin. Digested peptides were concentrated by evaporation and resuspended in 2% acetonitrile, 0.05% TFA. PEPotec^TM^ crude peptides for MEK1 WT (CNSPYIVGFYGAFYSDGE) and Y130C (CNSPYIVGFCGAFYSDGE) were synthesized with ^13^C(5),^15^N(1) isotopes on glutamic acid and carbamidomethyl cysteins (ThermoFisher Scientific). These isotope-labeled ‘heavy’ peptides were diluted 500× into peptide samples resulting from GluC/trypsin digestion. One third of each sample was analyzed by liquid chromatography-MS/MS on an Orbitrap Fusion mass spectrometer equipped with a nanoelectrospray ion source (ThermoFisher Scientific) and coupled to an UltiMate 3000 nanoRSLC chromatography system (Dionex). The system was operated in PRM mode. Briefly, peptides were trapped at 20 μl/min in 2% acetonitrile, 0.05% TFA on a 5 mm×300 μm C18 PepMap cartridge (Dionex). The pre-column was switch online upstream of a 50 cm, 75 μm Acclaim PepMap100 C18 column (Dionex), and peptides were eluted with a linear gradient of 5-40% solvent B (80% acetonitrile, 0.1% formic acid) over 30 min at 300 nl/min. MS2 spectra corresponding to the ‘heavy’ (synthetic) and ‘light’ (endogenous) targeted peptides were acquired during the whole gradient length using the XCalibur software version 3.0.63 (ThermoFisher Scientific). To achieve this, 1023.43(2+), 1026.43(2+), 1021.91(2+) and 1024.92(2+) m/z were successively isolated into the quadrupole analyzer in a window of 0.7 Da and fragmented by higher energy collision-induced dissociation (HCD) at 35% collision energy. The resulting fragments, in range of 120-2000 m/z, were detected by the orbitrap analyzer at a resolution of 30,000, with an automatic gain control target of 1e5 and a maximum injection time of 120 ms. Prior to each sample analysis, a blank run was performed using the same method (Fig. S2C,D).

The 10 most intense *b* or *y* fragment ions derivating from each parent mass were extracted from the raw files with Skyline software v3.6 (PMID 20147306) to allow the reconstruction of elution peaks. The superposition of fragments traces and the co-elution of ‘heavy’ and ‘light’ peptides were both utilized to confirm the detection of targeted peptides. Peptide quantification was performed using the area under the elution curve, as calculated via Skyline. For each peptide, the areas of the three most intense ions of the targeted endogenous peptide were summed and normalized relative to the area of the ‘heavy’ synthetic peptide (Table S2).

### MEF isolation and pERK induction

MEFs were obtained from E13.5 embryos, cultured and immortalized as described (Giroux et al., 1999). Cells were starved in medium containing 0.1% FBS overnight before treatment with 20% FBS, EGF or FGF2 at 2 ng/ml for 0, 3, 5, 10, 30 and 60 min. Protein extracts were prepared as described (Bélanger et al., 2003). Total protein lysates (20 µg) were resolved on a denaturing 10% SDS-PAGE and probed with rabbit monoclonal antibody against phospho-ERK1/2 (1/5000; Cell Signaling Technology), and with mouse monoclonal antibody against vinculin (1/5000; Sigma-Aldrich) as a loading control. The relative amount of phospho-ERK1/2 was obtained by densitometry analyses with the Fluor-S MAX MultiImager-captured images using ImageJ software. Experiments were performed in triplicate.

### Pulmonary stenosis histological analysis

Paraffin-embedded E13.5 embryos were sectioned at 4 μm. Morphology was analyzed following Hematoxylin and Eosin (H&E) staining. Pulmonary artery lumen surface area was measured every 8 µm along the left and right pulmonary arteries (∼20 measures per specimen).

### Skeletal analysis

Whole-mount skeletons were stained with Alcian Blue for cartilage and Alizarin Red for bone. Mice were first skinned, eviscerated and fixed overnight at 4°C in 95% EtOH. The next day, specimens were put into Alcian Blue 0.015%; 20% acetic acid prepared in 95% EtOH for 7 days at 37°C. Skeletons were then rinsed for 1 h in 95% EtOH, clarified overnight in 2% KOH and stained overnight in 0.003% Alizarin Red prepared in 1% KOH. Specimens were transferred in 1% KOH:glycerol (1:1) for 7 days, and kept at room temperature in glycerol:EtOH (1:1) until analysis.

### Brain immunostaining

Mice were anesthetized, transcardially perfused with 4% paraformaldehyde and postfixed. Brain tissue was extracted and vibratome sectioned for immunolabeling. Brain sections were incubated for 2 days with primary antibody: rabbit anti-GFAP (1:1000; Abcam ab7260), rabbit anti-Olig2 (1:1000; Millipore ab9610), mouse anti-NeuN (1:1000; Millipore MAB377) in 1× PBS 0.2% Triton with 5% normal donkey serum. Tissue was then washed 3× for 20 min in 1× PBS 0.2% Triton and incubated for 2 days in Alexa Fluor dye-conjugated secondary antibodies and DAPI. Confocal images of immunolabeled brain slices were acquired on a Zeiss LSM 800 confocal microscope. Three anatomically matched regions of primary sensory cortex and hippocampal CA1 from three independent sections were imaged per animal. Cortical and hippocampal regions of 1-2 mm^2^ were manually defined and assessed for the relative density of GFAP^+^ and Olig2^+^ cells. At least three mutants and three littermate controls were analyzed.

### Statistical analyses

Samples were statistically compared using the Student's *t*-test and analysis of variance on linear models (ANOVA), when appropriate. *P*<0.05 was considered statistically significant.

## Supplementary Material

Supplementary information

First Person interview
