# Supplementary material for: Mek1Y130C mice recapitulate aspects of human cardio-facio-cutaneous syndrome
Source: Dis Model Mech. 2018 Mar 1;11(3):dmm031278. doi: 10.1242/dmm.031278 (PMC5897723; doi:10.1242/dmm.031278)
Supplement: Supplementary information [file dmm-11-031278-s1.pdf]

© 2018. Published by The Company of Biologists Ltd | Disease Models & Mechanisms (2018): doi:10.1242/dmm.031278: Supplementary information

FIRST PERSON

First person – Rifdat Aoidi

First Person is a series of interviews with the first authors of a selection of papers published in Disease Models & Mechanisms, helping early-career researchers promote themselves alongside their papers. Rifdat Aoidi is first author on '*Mek1*^{Y130C} mice recapitulate aspects of human cardio-faciocutaneous syndrome', published in DMM. Rifdat conducted the work in this article while a PhD student in the lab of Jean Charron in Laval University, Canada. She is now a postdoctoral training fellow in Victor Tybulewicz's lab at the Francis Crick Institute, London, UK, working on analyzing the congenital heart defects in Down syndrome mouse model.

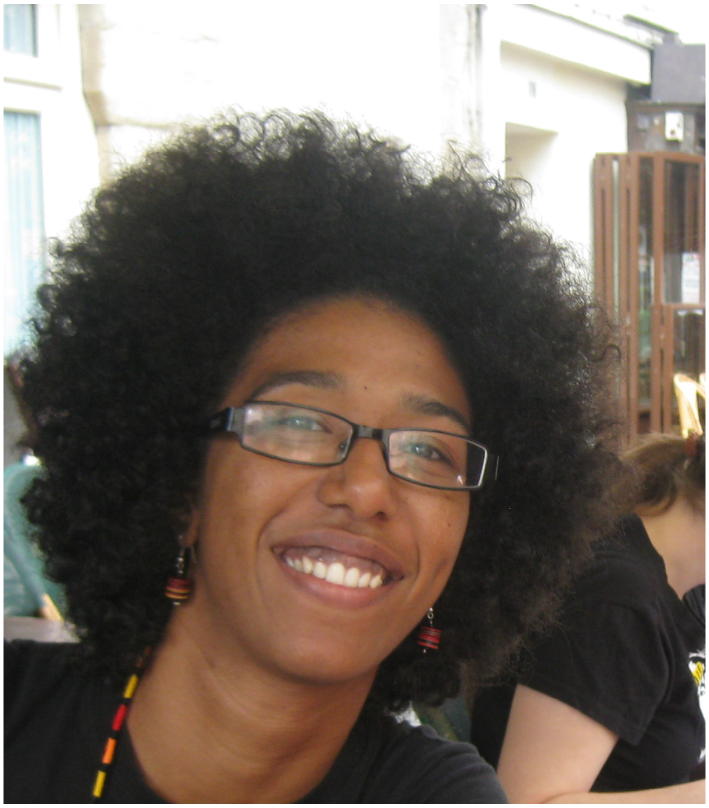

Rifdat Aoidi

How would you explain the main findings of your paper to non-scientific family and friends?

Cardio-facio-cutaneous syndrome (CFC) is part of the RASopathy, a group of rare genetic developmental syndromes. CFC syndrome is caused by mutations in genes called *BRAF* (~75% of patients), *MEK1* and *MEK2* (~25% of patients). These mutations result in a range of defects including craniofacial dysmorphology, cardiac malformations, cutaneous abnormalities and neurocognitive delay. We wanted to generate a mouse model in order to understand the syndrome and uncover the pathological mechanisms that lead to these defects. Knowing that among MEK1 and MEK2 mutations, MEK1^{Y130C} mutation is the most common, we decided to generate a mouse model carrying this mutation in order to investigate its molecular and developmental consequences. Analysis of these mice showed that Mek1^{Y130C} mutant mice exhibited pulmonary artery stenosis, cranial dysmorphia and neurological anomalies. Our data indicate that the Mek1^{Y130C} mutation recapitulates major aspects of CFC syndrome, providing a new animal model to investigate the physiopathology of this RASopathy.

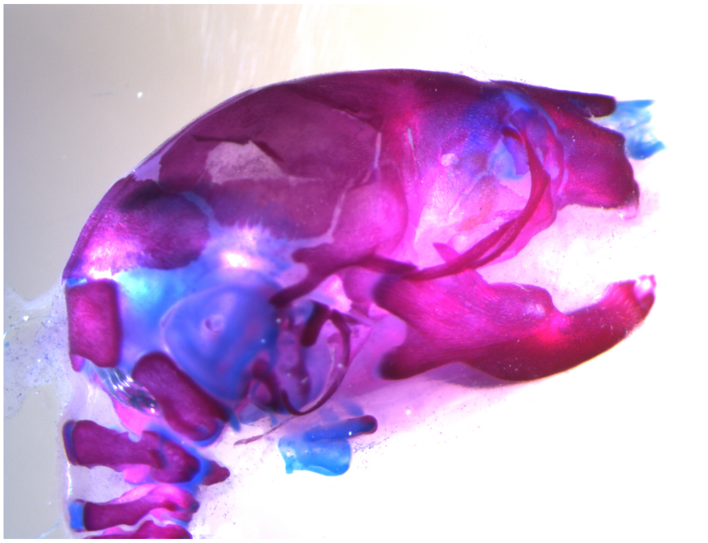

Newborn mouse head stained with Alcian Blue to show cartilage and Red Alizarin to show bones.

Rifdat Aoidi's contact details: The Francis Crick Institute, 1 Midland Road, London NW1 1AT, UK.

E-mail: rifdat.aoidi@crick.ac.uk

What are the potential implications of these results for your field of research?

To our knowledge, no mouse model carrying RASopathy-linked *Mek1* or *Mek2* gene mutations has been reported. Here, we generated the first mouse model for the most common MEK1 mutation, which gives a tool that can be used to analyze the mechanisms underlying the defects observed in CFC syndrome.

What are the main advantages and drawbacks of the model system you have used as it relates to the disease you are investigating?

CFC syndrome is a disorder associated with various developmental abnormalities. In order to investigate the causes and the mechanisms underlying defects observed in CFC syndrome, we have to analyze its development in genetically modified animals. Because of its high similarity with human in terms of anatomy, development and genetics, the mouse is the most frequently used mammalian model for human disease. I believe that the mouse is the most appropriate species because of the availability of the most developed genetic manipulation techniques and of the huge wealth of information about developmental biology. Mice are small, have a relatively short

lifespan and gestation time, and can be inbred allowing analysis in genetically homogenous strains. All of these features make them the species of choice to study human disease.

"Having a network of postdoctoral fellows can allow early career-scientists to meet more experienced postdocs in order to share our common experiences and discuss the issues that we all face."

What has surprised you the most while conducting your research?

Analysis of our *Mek1*^{Y130C} allele revealed that both wild type and Y130C mutant protein were expressed, and the MEK1^{Y130C} protein has lower expression that the wild type. These findings suggest that a duplication of the *Mek1* gene might have happened when generating the *Mek1*^{Y130C} allele. One of the strengths of our model is that despite reduced levels of MEK1^{Y130C} protein compared with wild type, *Mek1*^{Y130C} mutants showed increased activation of ERK (MAPK) in response to growth factors, supporting a role for MEK1^{Y130C} in hyperactivation of the RAS/MAPK pathway, leading to CFC. These findings are in agreement with previous findings suggesting that CFC mutations are gain-of-function that hyperactivate the RAS/MAPK pathway.

What changes do you think could improve the professional lives of early-career scientists?

For early-career scientists, the most challenging thing is to know to which funding bodies to apply in order to fund your research. Having a network of postdoctoral fellows can allow early-career scientists to meet more experienced postdocs in order to share our common experiences and discuss the issues that we all face.

What's next for you?

My long-term aim is to become an independent investigator studying human genetic diseases that result in developmental defects. To achieve this goal, I am currently undergoing postdoctoral training at the Francis Crick Institute in London, UK, working on analyzing the causes of congenital heart defects in Down syndrome mouse model.

Reference

Aoidi, R., Houde, N., Landry-Truchon, K., Holter, M., Jacquet, K., Charron, L., Krishnaswami, S. R., Yu, B. D., Rauen, K. A., Bisson, N., Newbern, J. and Charron, J. (2018). *Mek1*^{Y130C} mice recapitulate aspects of human cardio-faciocutaneous syndrome. *Dis. Model. Mech.* **11**: dmm031278.
